# Disrupted global metastability and static and dynamic brain connectivity across individuals in the Alzheimer’s disease continuum

**DOI:** 10.1038/srep40268

**Published:** 2017-01-11

**Authors:** Aldo Córdova-Palomera, Tobias Kaufmann, Karin Persson, Dag Alnæs, Nhat Trung Doan, Torgeir Moberget, Martina Jonette Lund, Maria Lage Barca, Andreas Engvig, Anne Brækhus, Knut Engedal, Ole A. Andreassen, Geir Selbæk, Lars T. Westlye

**Affiliations:** 1 NORMENT, KG Jebsen Centre for Psychosis Research, Division of Mental Health and Addiction, Oslo University Hospital & Institute of Clinical Medicine, University of Oslo, Norway; 2 Norwegian National Advisory Unit on Ageing and Health, Vestfold Hospital Trust, Tønsberg, Norway; 3 Department of Geriatric Medicine, The Memory Clinic, Oslo University Hospital, Norway; 4 Department of Medicine, Diakonhjemmet hospital, Oslo, Norway; 5 Department of Neurology, Oslo University Hospital, Oslo, Norway; 6 Faculty of Medicine, University of Oslo, Oslo, Norway; 7 Centre for Old Age Psychiatric Research, Innlandet Hospital Trust, Ottestad, Norway; 8 Department of Psychology, University of Oslo, Oslo, Norway

## Abstract

As findings on the neuropathological and behavioral components of Alzheimer’s disease (AD) continue to accrue, converging evidence suggests that macroscale brain functional disruptions may mediate their association. Recent developments on theoretical neuroscience indicate that instantaneous patterns of brain connectivity and metastability may be a key mechanism in neural communication underlying cognitive performance. However, the potential significance of these patterns across the AD spectrum remains virtually unexplored. We assessed the clinical sensitivity of static and dynamic functional brain disruptions across the AD spectrum using resting-state fMRI in a sample consisting of AD patients (n = 80) and subjects with either mild (n = 44) or subjective (n = 26) cognitive impairment (MCI, SCI). Spatial maps constituting the nodes in the functional brain network and their associated time-series were estimated using spatial group independent component analysis and dual regression, and whole-brain oscillatory activity was analyzed both globally (metastability) and locally (static and dynamic connectivity). Instantaneous phase metrics showed functional coupling alterations in AD compared to MCI and SCI, both static (putamen, dorsal and default-mode) and dynamic (temporal, frontal-superior and default-mode), along with decreased global metastability. The results suggest that brains of AD patients display altered oscillatory patterns, in agreement with theoretical premises on cognitive dynamics.

Dementia is a highly prevalent syndrome with a large economic and societal impact, characterized by the progressive impairment of cognition, behavioral changes and reduced quality of life^1^. Alzheimer’s disease (AD), the most common form of dementia, has complex and heterogeneous etiopathogenic underpinnings, including both genetic and environmental factors^2^. While some clinical, cognitive and neuropathological components of AD are widely acknowledged^1^,^3^,^4^, a unified frame for the intricate architecture of AD is still needed in order to make sense of the pathways from molecular changes to clinical phenomenology, which is critical for early identification of at-risk individuals.

Converging evidence suggests that large-scale brain oscillatory activity disruptions, frequently assessed using resting-state functional magnetic resonance imaging (fMRI), may reflect key mechanisms bridging the gap between neuropathology and cognitive alterations in AD^5^. Virtually all the resting-state fMRI literature on oscillatory alterations in AD has focused on measures of coupling between different brain regions averaged across a scanning session –the so-called (*static) functional connectivity*. For example, AD patients have been shown to exhibit stronger hippocampal connectivity within the default mode network and weaker hippocampal-cingulate co-activation^6^. Furthermore, suggesting that the clinical manifestation may be mirrored in preclinical stages in individuals with high genetic risk, young and middle-aged carriers of the AD-associated APOE-epsilon4 allele have shown higher hippocampal synchronization within the default mode network^7^,^8^. However, results across studies are largely mixed, suggesting both increases and decreases in coupling associated with aging and AD^9^.

Importantly, recent conceptual, theoretical and methodological advances may shed light on these mixed findings; among them, the observation that time-varying coupling and de-coupling of brain regions embeds information that may be important in clinical settings^10^,^11^. This novel and biologically feasible framework has been conceptualized in the *communication through coherence* theory, which postulates that cognition is linked to neural communication emerging from coherent oscillatory activity, and that cognitive functions require a flexible set of signaling processes occurring on top of the anatomical backbone of the brain^12^,^13^,^14^. Namely, in order to fully benefit from the fixed anatomical connections, neural ensembles should traverse distinct dynamic states for adequate cognitive and other mental functions to arise^15^,^16^. Consequently, brain disorders typically implicating cognitive and emotional functions are expected to be characterized by a reduced *dynamic repertoire*^17^: a smaller set of brain functional configurations. Of note, the underlying neurobiological mechanisms can take place at different timescales, including the very slow frequencies measured non-invasively via fMRI^15^, and can be measured by analyzing variations in synchrony among regions over time^18^,^19^.

In this study, we aim to test this conjecture by assessing the potential clinical sensitivity of brain oscillatory characteristics across the AD spectrum in a sample of patients with AD, individuals with mild cognitive impairment (MCI), and those with only subjective memory complaints (also known as subjective cognitive impairment, SCI). Time-series from functional “seeds” defined with independent component analysis (ICA) were estimated using dual regression^7^, and potential alterations in brain oscillatory activity were analyzed using two methods: 1) the standard, averaged coupling between pairs of resting-state fMRI signals^20^, and 2) phase-based metrics of coupling and de-coupling across time: *metastability* (whole-brain dynamics) and pairwise synchrony changes (dynamics of paired brain regions)^11^. Phase-based metrics of coupling have conventionally been used in electroencephalography studies, in measures such as the phase-locking value^21^,^22^,^23^, and have only recently been adapted for fMRI signal analysis of time-varying connectivity^10^,^11^,^24^. To the best of our knowledge, no previous study has evaluated this type of phase-based dynamic alterations in fMRI activity in an aging and neurodegeneration context. Based on established knowledge about the anatomical distribution of the pathophysiology and resting-state fMRI alterations in AD, and also considering theoretical concepts on the relevance of a wide set of time-varying connectivity, we hypothesized that patients with AD exhibit altered sFC and dFC, both at the whole-brain level and at specific brain nodes implicating temporal, parietal and subcortical structures, reflecting a graded pattern of differences corresponding with disease severity.

## Results

### Demographic and clinical data

**Figure 1:**
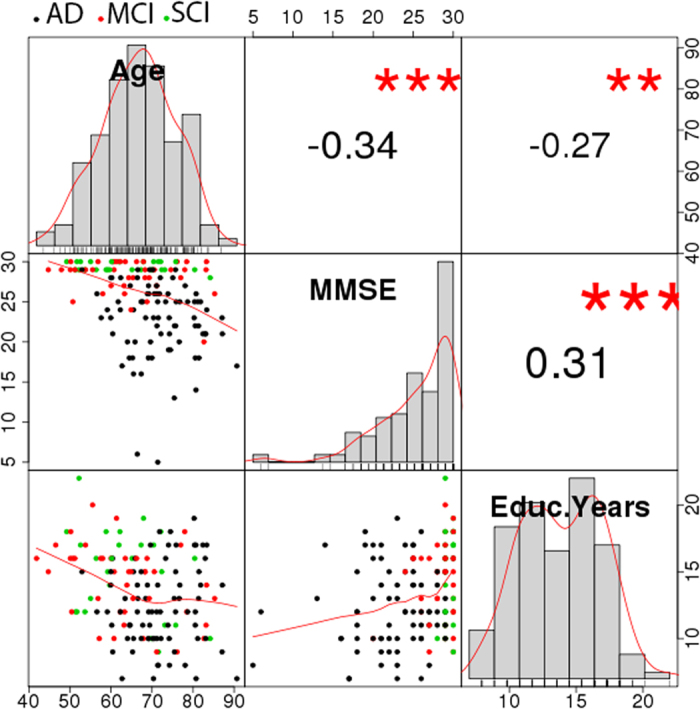
Correlation matrix chart of demographic features. Entries in the upper triangle correspond to Pearson’s correlation coefficients, and the number of “*” represents the significance level (**p < 10^−2^, ***p < 10^−3^).

The demographic data summarized in Table 1 and Fig. 1 shows statistically significant differences in the distribution of age, years of education and MMSE scores across the different diagnostic categories. Briefly, patients with AD (71.5 ± 7.9 years) were older than the MCI (63.5 ± 11.1) and SCI (63.6 ± 9.6) groups. Also, patients with AD had lower MMSE scores (22.8 ± 4.8) than MCI (28.2 ± 2.1) and SCI (29.4 ± 0.6), and shorter education. There were no differences in sex distribution between groups. The correlation coefficients between age, MMSE and education indicate small/medium effect sizes^25^, which justifies their inclusion as covariates without strong collinearity issues.

### Statistical analysis of average coupling (sFC)

**Figure 2:**
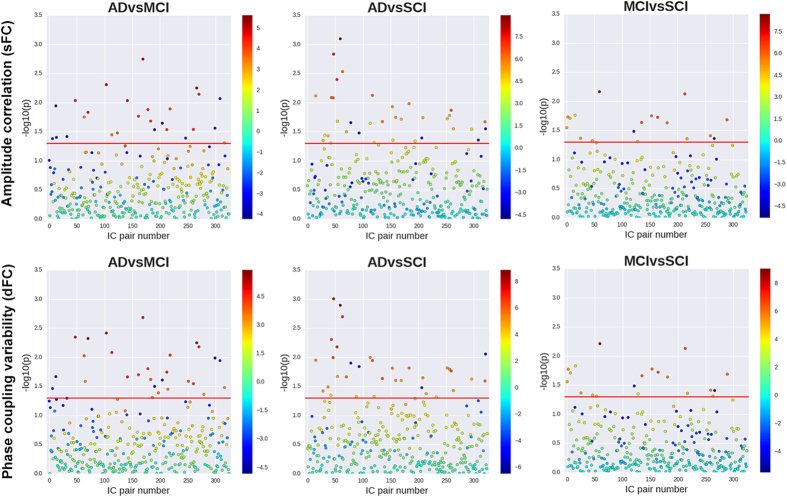
Logistic regression results for sFC and dFC at the 325 IC pairs. The results represent raw *p*-values were obtained from logistic regression models adjusting by gender, age, in-scanner motion and headcoil. The horizontal red line indicates the *p*-value threshold (0.05) in the corresponding logarithmic scale. Colorbars display regression coefficients for the different pairs.

**Figure 3:**
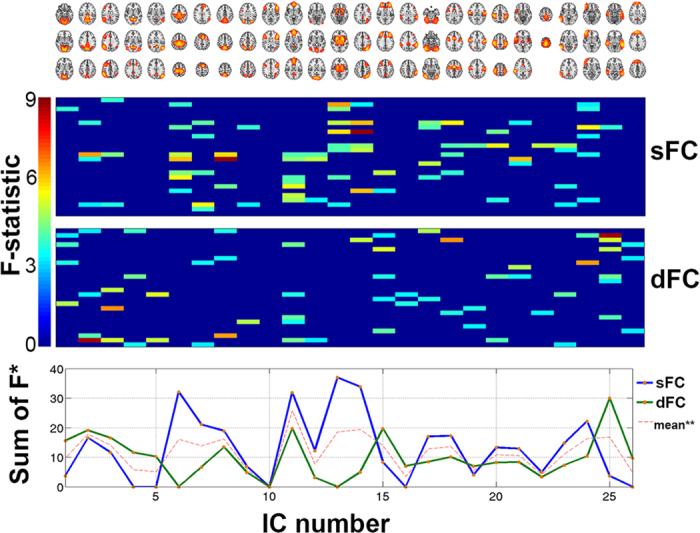
IC pairs in the statistically significant sub-networks (sFC and dFC) from NBS. Heatmap matrices: the entries represent F-statistics of the 28-edge network with NBS significance. Non-significant entries were set to 0. Notes: *sum of F-statistics from the statistically significant results, illustrating the relative importance of each IC; **average of “sum of F” over dFC and sFC.

Figure 2 (top) displays regression coefficients and unadjusted *p*-values obtained from logistic regression analysis of diagnosis pairs (ADvsMCI, ADvsSCI and MCIvsSCI), using each of the 325 partial correlation coefficients as independent variable, and controlling for gender, age, headcoil and motion. Network-based statistics (NBS) analysis using data from all participants showed a 39-edge subnetwork with diagnose-dependent disruptions (*p* = 0.037) (Fig. 3, uppermost heatmap). As shown by the sum of F-statistics in Fig. 3 (bottom), the brain nodes with the largest contributions to the statistical significance of that subnetwork were IC13 (putamen), IC14 (dorsal), IC11 (default-mode) and IC6 (default-mode).

**Figure 4:**
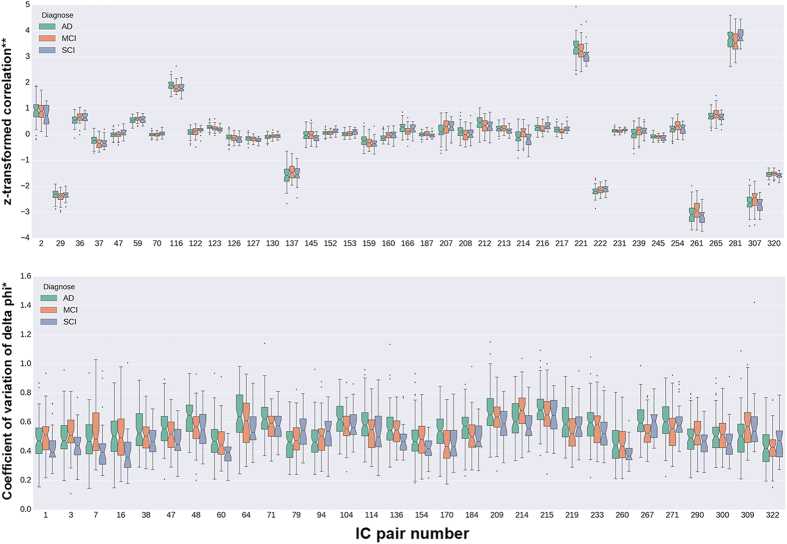
Raw sFC and dFC values at the IC pairs with significance from the NBS tests. The trends shown here corresponds to the direct measures of pairwise coupling (see *Methods*), whereas the test results discussed in the text show that the association between diagnostic status and dFC persists even after adjustments for gender, age, and headcoil. Notes: *coefficient of variation (standard deviation divided by mean) of the normalized phase differences (see *Methods*); **z-transformed value of the regularized regression coefficients.

Figure 4 (top) shows the partial correlation coefficients (z-transformed) of the 39 edges constituting the mentioned network. Notice that the plot contains unadjusted data, which are not directly comparable to the F-statistics derived from NBS multivariate tests; visual analysis of the boxplot notches (namely, non-overlapping notches as evidence of median differences^26^) indicates that the largest between-group differences are in the following IC pairs: 36 (IC2-13: 0 < AD < MCI = SCI), 116 (IC6-7: 0 < MCI = SCI < AD), 137 (IC7-9: AD < MCI = SCI < 0), 160 (IC8-14: AD < MCI = SCI = 0), 166 (IC8-20: AD > MCI = SCI > 0), 207 (IC11-13: 0 < AD < MCI = SCI), 214 (IC11-20: AD = SCI < 0 < MCI), 216 (IC11-22: 0 < MCI < AD = SCI), 217 (IC11-23: 0 < MCI < AD = SCI), 222 (IC12-14: AD < MCI < SCI < 0), 239 (IC13-18: 0 = AD < MCI = SCI), 254 (IC14-21: 0 < AD = SCI < MCI), 261 (IC15-17: 0 < MCI < AD = SCI), 265 (IC15-21: 0 < AD = SCI < MCI), 281 (IC17-18: 0 < MCI < AD = SCI) and 307 (IC20-23: 0 < MCI < AD = SCI). Notice that, from the previous list, IC pairs 137, 222, 261, 281 and 307 show, on average, large z-transformed correlations (absolute value > 1), and all other IC pair coefficients were reduced via regularized partial correlation, as explained in *Methods*. These results indicate the presence of both increases and decreases in functional coupling which could discriminate between diagnostic categories.

### Statistical analysis of time-varying connectivity (dFC)

The 325 dFC measures were largely uncorrelated with each other, and showed only weak correlations with their sFC counterparts (Figure S1), further justifying their inclusion as alternative phenotypes. Coefficients and *p*-values of the different IC pairs, derived from logistic regression tests over pairs of diagnoses, are shown in Fig. 2 (bottom). While most of the test statistics at *p* < 0.05 indicate decreased dFC with increased disease severity (negative-valued coefficients depicted as dark blue dots), none of these results survives a stringent Bonferroni correction. However, after multiple-testing adjustments using NBS on the three-diagnose data, there was a 30-edge dFC sub-network with alterations related to disease severity (*p* = 0.018) (Fig. 3, lowermost heatmap). The brain regions with the largest contributions to the statistical significance were related to the temporal/auditory (IC 25), frontal (IC 15) and default-mode (IC2 and IC11) areas.

The dFC values (coefficients of variation) at the 28 altered edges, grouped by diagnosis, are shown in Fig. 4 (bottom). As mentioned for the case of sFC, Fig. 4 displays unadjusted data, which are not directly comparable to the F-statistics derived from NBS multivariate tests; nevertheless, the different distributions of dFC values depending on diagnosis can be appreciated. Visual analysis of boxplot notches^26^ suggests that the strongest between-group dissimilarities observed in the raw data indicates localized decreases in dFC with increasing disease severity. More specifically, the following IC pairs showed that pattern: 1 (IC1-2: SCI < AD < MCI), 16 (IC1-17: SCI < MCI < AD), 48 (IC2-25: SCI = MCI < AD), 60 (IC3-14: SCI < MCI < AD), 64 (IC3-18: SCI < MCI < AD), 71 (IC3-25: SCI = MCI < AD), 79 (IC4-11: AD < MCI = SCI), 94 (IC4-26: AD < MCI < SCI), 136 (IC7-8: SCI < MCI = AD), 170 (IC8-24: SCI = MCI < AD), 184 (IC9-21: SCI = MCI < AD), 214 (IC11-20: SCI = AD < MCI), 233 (IC12-25: SCI = MCI < AD), 267 (IC15-23: MCI < SCI = AD), 271 (IC16-17: MCI < SCI = AD), 309 (IC20-25: AD < MCI = SCI) and 322 (IC23-26: AD = MCI < SCI). These results thus suggest that different patterns (both increases and decreases) of localized dFC may be related to diagnose.

**Figure 5:**
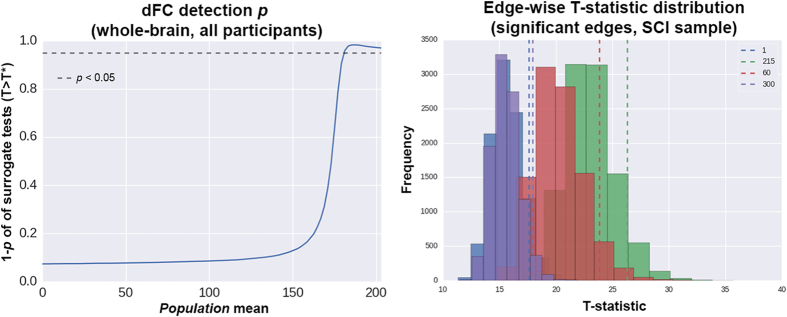
Surrogate data test results at the whole-brain level and at edges with group differences. Left: test of the sampled dFC values (sum of 325 edge weights) against a range of population means. Right: analysis of the dFC empirical values against their surrogate null distributions in the SCI group. The plot shows only the four edges with both statistically significant (clinical) group differences and p < 0.1 in the surrogate tests. Numbers next to the dashed line legends correspond to IC pairs. Additional details on the test procedures are described in *Results*.

**Figure 6:**
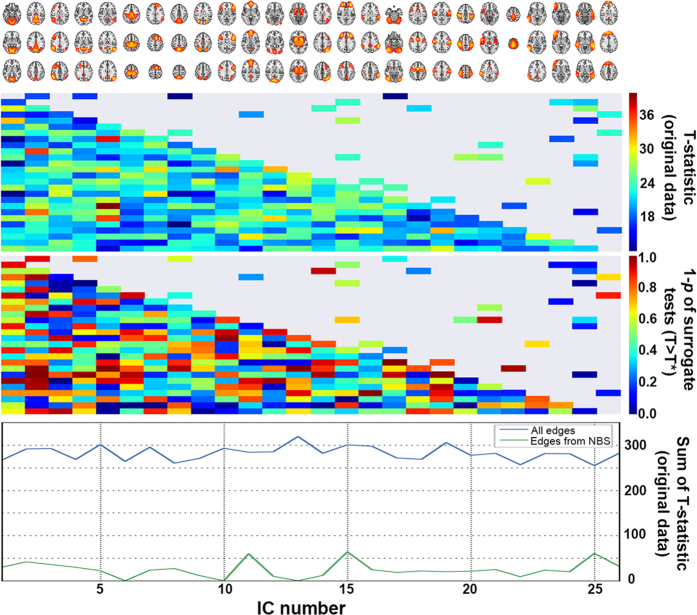
Results of surrogate data tests for dFC in the SCI group. Uppermost heatmap: T statistic maps from one-group T-tests (null hypothesis: dFC population mean = 0 at every edge). Only the original data was used for that plot. The upper diagonal shows only connections with statistically significant differences depending on clinical diagnose. Lowermost heatmap: using 10000 surrogate datasets, T* values were estimated, and 1 minus the proportion of times with T (original) > T* (surrogate) was considered the *p*-value for the presence of true dynamics. Note that the colormap corresponds to 1-*p* (lower *p*-values have warmer colors). In both heatmaps, the upper diagonal shows only connections with statistically significant differences depending on clinical diagnose. Bottom: sum of (empirical) T-statistic for each IC.

The analysis of surrogate data showed that, in the present setting, the probabilities of detecting weak dFC are small. As shown in Fig. 5, in the whole dataset, the detection probabilities are above chance level only in the presence of strong dFC. For the specific case of the connections with (clinical) group differences, the strongest evidence for temporal dynamics at pairs 300 (IC19-22, *p* = 0.038), 60 (IC3-14, *p* = 0.062), 215 (IC11-21, *p* = 0.078) and 1 (IC1-2, *p* = 0.092) (Figs 5 and 6), suggesting that the current approach might have moderate power to detect weak dFC at such edges. As inferred from the data (Fig. 6), none of those results would be statistically significant when applying multiple testing adjustments; however, such corrections may be overly conservative here since the assessed edges come from another analysis, which already implemented a network-based multiple testing protocol. Since the evidence of true temporal dynamics observed here is only moderate, it should be noted that the group differences (AD/MCI/SCI) might have been influenced by non-dynamic properties of the time series.

### Time-varying synchronization at the whole-brain level: metastability

**Figure 7:**
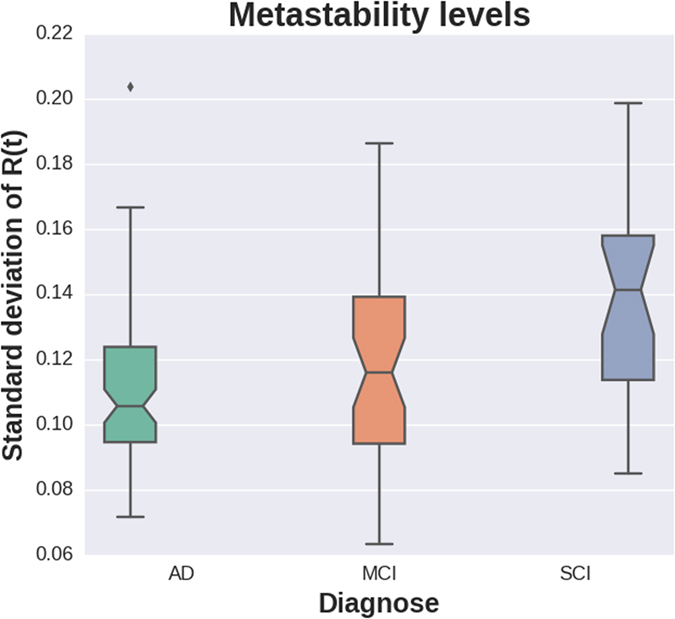
Association between metastability and diagnostic status. Note: The trend shown here corresponds to the direct measures of metastability, whereas the test results discussed in the text show that the association between diagnostic status and metastability persists even after adjustments for gender, age and headcoil.

After adjusting for potential confounders, whole-brain metastability levels were higher in the healthier subgroup (SCI), and progressively decreased through MCI and AD (multinomial logit with SCI as reference group: β_MCI_ = −25, SE_MCI_ = 9.1, *p*_MCI_ = 0.006; β_AD_ = −29.1, SE_AD_ = 8.9, *p*_AD_ = 0.001; pseudo-*R*^2^ = 0.154, log-likelihood = −126.8, Fig. 7). Of note, neither the raw motion estimates obtained with MCFLIRT nor the variance kept after filtering were related to that metastability metric, and there was no evident movement*diagnose interaction associated with metastability (Figures S2 and S3). There was no association between metastability and tSNR, neither before nor after running FIX (linear regression metastability~tSNR: before FIX: β = −1.4 * 10^−4^, SE = 1.1 * 10^−4^, *p* = 0.217; after FIX: β = −1.7 * 10^−4^, SE = 1.1 * 10^−4^, *p* = 0.223; Figure S2). Similarly, there was no significant interaction with diagnosis (linear regression metastability~tSNR*diagnosis, with AD as reference group: before FIX: β_MCI_ = 6 * 10^−5^, SE_MCI_ = 2.6 * 10^−4^, *p*_MCI_ = 0.817, β_SCI_ = −2.2 * 10^−4^, SE_SCI_ = 3.2 * 10^−4^, *p*_SCI_ = 0.495; after fix: β_MCI_ = 1.6 * 10^−4^, SE_MCI_ = 3.1 * 10^−4^, *p*_MCI_ = 0.603, β_SCI_ = 9.6 * 10^−5^, SE_SCI_ = 4.7 * 10^−4^, *p*_SCI_ = 0.838; Figure S3).

## Discussion

The present study evaluated the potential association between fMRI based neural oscillatory coupling disruptions and clinical severity across a phenotypical continuum related to cognitive status (AD, MCI and SCI). Two different types of between-region oscillatory coupling were analyzed: the average across the scanning session (*static*, sFC), defined as the regularized partial temporal correlation between pairs of nodes, and between-region oscillatory coupling was measured as the instantaneous transitions between synchronization and de-synchronization (dynamic, dFC), defined as the coefficient of variation of the phase differences over time. Distinct group differences in sFC and dFC were observed. Additionally, a statistically significant pattern of whole-brain metastability alterations was observed, with AD showing lower values than MCI, and MCI displaying lower values than SCI.

The findings on static coupling data (sFC) indicate between-group differences in coupling patterns mainly implicating the putamen, dorsal and default-mode areas, indicative of either hyper- or hypo-synchronization at specific regions depending on diagnosis. This is in line with previous data on the anatomical location of neurodegenerative and AD-related changes, and also resembles the accumulated literature findings of both increases and decreases in functional connectivity associated with aging and AD^27^.

To our knowledge, this is the first report on metastability and related phase coupling alterations (dFC) measured with fMRI in AD-spectrum phenotypes. The results agree with recent theories on brain computation, which posit metastability as the optimal state of neural activity at rest: a dynamical regime characterized by rapid, flexible, engaging and disengaging neural ensembles^16^,^28^,^29^,^30^. Within that framework, higher metastability at rest is thought to underlie optimal cognitive-behavioral function^28^,^31^; the findings here suggest that metastability gradually decreases from the healthier (SCI) to partly affected (MCI) and more severely altered cognitive states (AD). It is also worth noting that the dynamic differences measured globally (whole-brain metastability) were accompanied by localized disruptions in dFC, implicating a wide range of brain network nodes and their connections, suggesting a key role for temporal, frontal and default-mode areas in AD dFC.

The relevance of the regions and brain networks found using sFC and dFC (including default-mode, frontal, putamen, dorsal and temporal areas) has been highlighted in former research on brain changes throughout the AD continuum, such as functional alterations detected with resting-state fMRI^32^,^33^,^34^,^35^, volumetric atrophy observed with MRI^36^,^37^, and amyloid neuropathological disturbances measured with positron emission tomography^38^. Jointly, these results expand on a system-wide view of AD dementia^5^,^39^, by endorsing that the anatomically-constrained AD neuropathology may be linked to some localized macro-scale brain function alterations ultimately leading to the clinical phenotypes.

Two behavioral fMRI reports on humans deserve discussion in light of the present dFC results. In perhaps the most similar study to this one, Hellyer *et al*.^28^ investigated cognition and metastable dynamics in a sample of 89 participants (either healthy or with traumatic brain injury); they found that reduced metastability (as measured with resting-state fMRI) was associated with structural connectivity damage, reduced cognitive flexibility and disrupted information processing. Added to the previous findings, this may suggest that altered resting-state dynamics are a landmark of neurocognitive alterations across distinct clinical phenotypes. Furthermore, in a study using sliding-window analysis, Sourty *et al*.^40^ reported that some brain function alterations in Lewy body dementia can be detected using dFC but not static functional connectivity. Importantly, measuring dFC might have been troublesome in that report due to the low statistical detection power of single-session sliding-window correlations^18^. Novel methodologies, such as the phase-synchrony approaches adopted in this study^10^,^24^, might help overcome those issues. Here, they allow expanding on the previous clinical findings in dementia with Lewy bodies^40^ by suggesting that dFC alterations can be found in another form of dementia (AD) when measuring phase differences.

Finally, it is worth highlighting that the observed metastability decreases in AD might be related to a shrinkage of the *dynamic repertoire*, which constitutes a candidate physiological mechanism to connect classical neuropathological and clinical observations, and can be interpreted in that context. For instance, neuropathological findings have shown that aging, AD and amyloid alterations are associated with key myelin aberrations^41^,^42^,^43^,^44^. In parallel, research combining theoretical neuroscience and resting-state fMRI measurements experiments indicates that the dynamic patterns of the brain are constrained in cases of degradation of myelination, due to a restricted capacity of the neural ensembles to change its spatial configuration (transmission speed restrictions)^17^. Namely, micro-scale alterations of axonal conduction velocities –commonly due to myelin pathology– are thought to underlie macro-scale dFC reductions observed with fMRI. Additionally, at the psycho(patho)logical level, the efficient dynamical processing (over time) of neural information by segregating and integrating information is thought to sustain flexible cognitive states^16^,^31^,^45^,^46^, making fMRI-based metastability and dFC decreases feasible candidate indices of neurocognitive pathology. The metastability-related alterations observed in patients with AD fit well into biological and clinical frameworks, to suggest that large-scale brain oscillatory disruptions may link micro-scale neural alterations with disrupted cognitive architectures reported from the clinical phenomenology, which may eventually inform new therapy development using pathoconnectomics^47^.

Some limitations should be noted. First, the moderate sample size could have restricted the power to detect some effects. However, the cases where statistically significant outcomes were detected may indicate the presence of relatively large population effect sizes. Secondly, potential clinical heterogeneities across participants might affect the consistency of the findings: different subtypes of SCI and MCI may be associated with considerable differences in risk for dementia^48^,^49^, and probably also with distinct brain characteristics (i.e., Nobili *et al*.^50^). Robust neuroimaging findings from new studies may help address this issue; also, data on genetic and neuropathological markers might allow better diagnostic classifications. Additional studies might take advantage of those markers, which may be better suited for study within larger samples that allow improved phenotypic (diagnostic) stratification. Furthermore, some clinical data indicates that SCI and MCI could be relatively close transitional states preceding AD^51^,^52^,^53^, which may be reflected as similar brain dynamics in the two diagnoses. Although there were robust statistical associations, the lack of a healthy control group that had not been referred from the memory clinic may have limited the power to detect some MCI- and AD-specific effects. Even though the category of SCI is still controversial and may lack clinical relevance, some reports indicate that it could be associated with minor brain changes^54^. Hence, future studies may benefit from the inclusion of healthy controls without cognitive complaints to improve specificity, and prospective longitudinal designs are needed to assess the predictive value of the sFC, dFC and metastability indices in a clinical dementia context. Besides, even though stringent image pre-processing methods were applied, the influence of artifacts cannot be completely ruled out. Moreover, since the relationship between neural responses and blood-oxygen-level dependent fMRI signals has not been fully elicited^55^,^56^, and also considering the issues associated with causation analysis in fMRI^57^, complementary experiments are needed to explain the ultimate pathological mechanisms behind the observed patterns. Finally, as discussed by Glerean *et al*.^24^, the phase synchrony methods used to estimate dFC have some limitations: for instance, the current results correspond to the chosen narrowband (0.04–0.07 Hz), but other frequency domains could contain complementary information; also, the amplitude envelope is not used, even though it might complement the analyzed phase metrics. It is also worth mentioning that the ongoing dFC approach offered moderate evidence of temporal dynamics within the dataset considered here. This observation emphasizes the importance of conducting surrogate data tests –or equivalent– in order to verify the presence of dynamics in fMRI signals. That being said, having detected a different set of relevant alterations in AD when using sFC and the current dFC metrics suggests that they might inform on complementary pathophysiological mechanisms.

Overall, the results reported here constitute empirical evidence on relatively unexplored neural activity disruptions through different cognitive impairment phenotypes related to neurodegeneration, which are in line with previous clinical and neuropathological observations of an AD continuum^52^,^54^,^58^,^59^,^60^. Notwithstanding the limitations of this work, the observed metastability, sFC and dFC patterns are in agreement with the literature^31^ and deserve further attention across various cognitive and clinical phenotypes.

## Methods

### Ethics statement

The Regional Committee for Medical Research Ethics in South-Eastern Norway approved the study. Patients were only enrolled if determined to have capacity for consent by the evaluating physician. All participants gave written informed consent. All procedures were conducted in accordance with the Helsinki Declaration.

### Participants

Cross-sectional patient data were obtained from the “Norwegian registry for persons being evaluated for cognitive symptoms in specialized care (NorCog)”. NorCog is a national patient registry comprising consecutively enrolled patients referred to one of 27 participating memory outpatient clinics because of suspected cognitive impairment or dementia.

All patients in the present study were recruited from one of the NorCog centers, the memory clinic at Oslo University Hospital, between 2010 and 2014. Patients were assessed in accordance with a standardized examination protocol^61^. Patients at the Oslo University Hospital memory clinic are usually referred to MRI of the brain as part of the diagnostic workup. Between 2010 and 2014 a limited capacity research protocol MRI was available. Selection of referral to this alternative was only based on accepted waiting time. The patients were diagnosed by two doctors in consensus (KE/AB or MLB/KP), based on all available information from the extensive clinical examination. Only patients with AD (n = 80, 71 ± 8 years, 35 males) according to the ICD-10 criteria^62^, patients with MCI (n = 44, 64 ± 11 years, 26 males) according to Winblad criteria^63^, and patients with a subjective cognitive complain that did not fulfill the AD or MCI criteria (SCI, n = 26, 64 ± 11 years, 26 males)^64^, were included in this study. The results of the Mini-Mental State Examination (MMSE, Norwegian version) from the clinical assessment were used as a measure of cognitive impairment^65^,^66^. Additional descriptive information is summarized in Table 1 and Fig. 1.

### Image acquisition

T2*-weighted MRI was obtained on a 3 T General Electric Signa HDxt with different head coils (8-channel, HDNV and HNS), using an echo planar sequence with 203 volumes and the following parameters: repetition time (TR) = 2638 ms; echo time (TE) = 30 ms; flip angle = 90°; acquisition matrix = 64 × 64; in-plane resolution = 4 × 4 mm; 45 axial slices; slice thickness = 3 mm. Each dataset comprised 202 volumes, summing up a total of 202 × 2.638 s = 532.876 s (8 minutes, 53 seconds). For technical reasons, three different head coils were used: standard GE 8-channel (57 AD, 34 MCI and 19 SCI), HDNV (1 AD and 1 SCI) and HNS (21 AD, 10 MCI and 6 SCI). Fisher’s exact test for contingency tables did not reveal significant differences in the distribution of participants across headcoils (two-tailed *p* = 0.708). T1-weighted data was also collected, and used for co-registration purposes in the current study (FSPGR sequence; TR = 7800 ms; TE = 2.956 ms; TI = 450 ms, flip angle = 12°; in-plane resolution = 1 × 1 mm; number of sagittal slices = 166; slice thickness = 1.2 mm; acquisition time = 7 min 8 s).

### Image pre-processing

fMRI data pre-processing was conducted using diverse tools of the FMRI Expert Analysis Tool from the FMRIB Software Library (FSL)^67^,^68^,^69^. Pre-processing steps included brain extraction, motion correction, spatial smoothing (Gaussian kernel, full-width at half-maximum = 6 mm) and high-pass filtering (100 s). The distribution of estimated mean relative motion (volume-to-volume displacement) obtained with FSL’s MCFLIRT is shown in Figure S4. The estimated mean relative in-scanner head motion measured with FSL’s MCFLIRT differed among diagnostic groups, due to a few AD participants with high movement rates (Kruskal-Wallis rank sum test *X*^2^ = 6.15, 2 degrees of freedom, *p* = 0.046; Figure S4). Mean relative motion was later used as a covariate in the statistical analyses. FMRIB’s ICA-based Xnoiseifier (FIX)^70^,^71^ was used for data denoising, with a conservative threshold of 60. Briefly, ICA is applied on single-subject data, and the resulting components are submitted to classification as “noise” or “signal” by comparison with a standard catalogue of independent components. Components classified as “noise” are then regressed out. Based on the temporal signal to noise ratio (tSNR)^72^ computed on the denoised data, three participants were discarded (initially, *n* = 153). Before FIX, there were between-group differences (Kruskal-Wallis rank sum test: *X*^2^ = 7.6, 2 d.f., *p* = 0.022; Table S1), due to lower tSNR in the SCI subset. Of note, those between-group differences in tSNR were removed after FIX (Kruskal-Wallis rank sum test: *X*^2^ = 2.7, 2 d.f., *p* = 0.259; Table S1).

To obtain precise brain masks for fMRI co-registration, automated whole-brain segmentation was performed on the T1-weighted anatomical references in FreeSurfer^73^,^74^; the surface reconstructions were visually inspected and manually edited when necessary. The fMRI datasets were then registered to the individual structural scan using FLIRT and boundary-based registration^68^,^75^. The structural scan was warped to the Montreal Neurological Institute MNI152 template (2 mm) using FNIRT^75^,^76^,^77^, before applying the same warping to the fMRI data.

**Figure 8:**
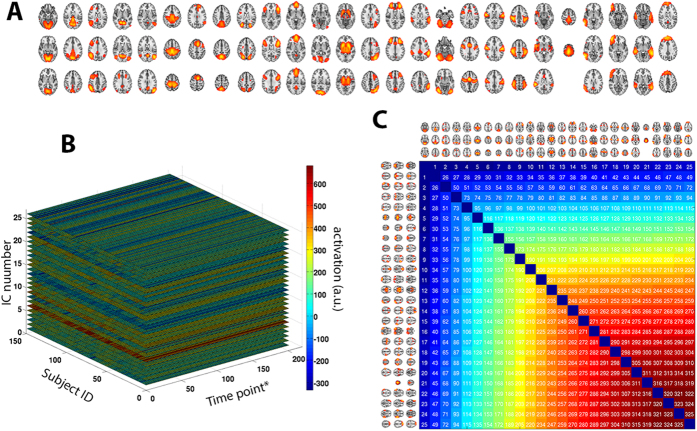
Parcellation-free spatial maps obtained using ICA. (**A**) After applying an automatic model order selection and manually removing potential artifacts, 26 spatial components remained. (**B**) Activation levels derived from fMRI were set into a matrix of [150 subjects]×[202 time points]×[26 components]. Abbreviations: *, sampling time point, corresponding to the TR (here, 2638 ms); a.u., arbitrary units (of activation intensity). (**C**) Anatomical reference for IC pairs: each pair of ICs was assigned an arbitrary number, which is used as reference in the manuscript.

Next, in order to avoid bias in the group-level ICA, three balanced subsets of participants were formed (26 AD, 26 MCI and 26 SCI; no significant differences in gender and age), as proposed by Kaufmann *et al*.^78^; an automatic estimation of model order generated 34 ICs. Individual component spatial maps and corresponding time-series were obtained for each subject in the full sample (*n* = 150) by means of dual regression^7^. Following standard procedures^79^, eight components were removed after visual screening, giving a total of 26 ICs’ time-series included in the final analyses. Additional information about those 26 ICs is shown in Fig. 8.

### Average coupling of fMRI signals: *static functional connectivity (sFC*)

The 26 time-series mentioned above were then submitted to further processing using FSLNets^20^, running under Matlab (The Mathworks Inc.). In short, the pairwise coupling of these signals (a unique session-wide average per pair) was obtained by L1-norm regularization of the estimated inverse covariance matrix^78^,^80^, in order to force to zero the small and potentially noisy values. A total of 325 unique regularized partial correlation measures was obtained and standardized using Fisher’s z transformation; each pairwise correlation is typically considered a functional connection. In the present context, such statistical relationship is referred to as *sFC (static functional connectivity*), in contrast to the time-varying dynamic patterns described below.

### Phase-based metrics of time-varying patterns: *dynamic functional connectivity (dFC*)

**Figure 9:**
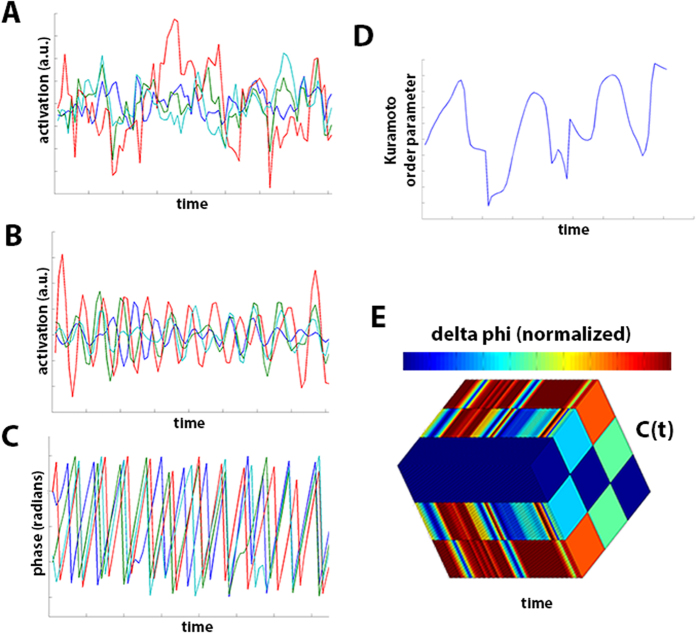
Computation of phase-based coupling parameters. The pictures exemplify computation of pairwise phase-based coupling and metastability, for data from 4 ICs (one participant). (**A**) Four time series collected using FSL’s ICA pipelines. (**B**) The time series are band-pass filtered (0.04–0.07 Hz), to later compute the Hilbert transform. (**C**) Individual phases obtained from the imaginary component of the Hilbert transform. (**D**) Kuramoto order parameter estimated across time using the phases. (**E**) Pairwise phase-coupling matrices, C(t), estimated for the 4 phases, across all time points; delta phi (Δ*ϕ*) is normalized as described in *Methods*.

Two different metrics were computed to measure dFC. For whole-brain, *metastability* was measured by calculating the standard deviation of the Kuramoto order parameter (an index of oscillatory coupling of all regions at every instant; see Fig. 9). A wide range (large standard deviation) of Kuramoto order parameter values would thus characterize brains that traverse different dynamic stages of coupling over time: a broad set of dynamic states, measured as high *metastability*. Additionally, for pairs of brain regions, the level of fluctuation between synchronization and de-coupling across the scanning session was estimated as the normalized differences in their wave phases.

The specific procedure was conducted as follows. The 26 time-series obtained from ICA and dual regression were further processed in Matlab (The Mathworks Inc.) to estimate metrics of time-varying connectivity across the scanning session, based upon relevant literature^10^,^11^,^24^. First, each series was narrow-band filtered within 0.04–0.07 Hz, which is required to later obtain meaningful phases^24^. Then, the Hilbert transform was applied by computing, for each time-series *x*(*t*),


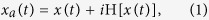


where H[·] stands for Hilbert transform and 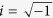. *x*(*t*) can also be expressed as an amplitude-modulated signal *a*(*t*) with carrier frequency *φ*(*t*), so that


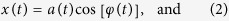



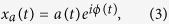


where *a*(*t*) is the instantaneous envelope and *ϕ*(*t*) the instantaneous phase. As suggested by Ponce-Alvarez, *et al*.^11^, the first and last ten time points were removed to minimize border effects from the Hilbert transform. The instantaneous phase values are computed for each of the 26 ICs, and then the Kuramoto order parameter (a proxy for the instantaneous whole-brain synchronization) is estimated as:


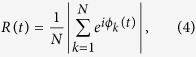


where *k* = {1, …, *N*} stands for IC number (here, *N* = 26), and *ϕ*_*k*_(*t*) is the instantaneous phase of the *k*-th oscillator (time-series) at time *t*. The *metastability* was then calculated as the standard deviation of *R*(*t*)^15^. Additionally, pairwise estimates of dynamic phase (de)coupling are estimated as follows: for each time instance *t*, the pairwise difference in phase between the time-series *i* and time series *j* is computed as:


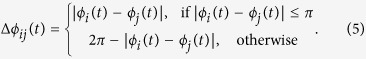


For each *t*, an instantaneous coupling matrix *C*(*t*) is obtained by normalizing those phase differences as 1 − Δ*ϕ*_*ij*_/*π*. The coefficient of variation (standard deviation divided by mean) of each element *C*_*ij*_(*t*) across all time points is the final pairwise metric of phase coupling/de-coupling. From here, there are 325 unique values in the matrix representing relationships between couples of time-series - which can be considered measures of dFC. The whole procedure is schematized in Fig. 9.

This approach was originally introduced by Glerean *et al*.^24^, who provided evidence that phase synchronization from fMRI signals can convey reliable information on time-varying connectivity; it was later adapted by Demirtas *et al*.^10^; in both cases, surrogate data was generated to test for the presence of dynamic patterns using previously described randomization procedures^81^,^82^. Considering the set of narrowband fMRI time-series as an ensemble of chaotic oscillators^83^,^84^,^85^, these techniques are based on the observation that phase synchronization can be measured from their Hilbert-transformed signals^86^.

To assess whether, in the current dataset, the coefficient of variation of *C*_*ij*_(*t*) can be considered a measure of temporal dynamics, statistical tests were conducted using surrogate data. Briefly, as recommended by Prichard and Theiler^82^, multivariate time series of the phase-randomized Fourier transform were obtained using the original dataset; a total of 10000 surrogates was generated, mimicking both the autocorrelations of each variable (IC signal) and the cross correlations between all variables. This simultaneous phase randomization method was implemented using publicly available Matlab code (https://se.mathworks.com/matlabcentral/fileexchange/32621-phase-randomization/content/phaseran.m) with the unfiltered time series; then, after filtering each of the 10000 datasets separately, the coefficient of variation matrix of *C*_*ij*_(*t*) was computed. The evidence of temporal dynamics was then evaluated at both the whole-brain level (by averaging all individual dFC values of each subject) and for single connections retrieved from the inter-group results. First, the sum of all unique dFC values was estimated for every participant as a general measure of dFC, and a series of one-sample T-tests was performed to evaluate whether the sampled dFC values (in the 150 participants) were drawn from a population with mean dFC ranging from 0 (weak dFC) to the maximum observed value in the surrogates (strong dFC). This set of T statistics was compared with the surrogate T* metrics, and a *p*-value was obtained for each of the assessed population means as 1 minus the fraction of times that T > T*. Additionally, similar tests were performed for single connections, with a special focus on the edges with statistically significant differences at the group level. Briefly, for each dFC metric (connection) in the original data, a one-sample T-test was conducted to evaluate whether in the 26-participant SCI group (the healthiest participants in this study, where the dFC signal should not be confounded by brain alterations), the sampled dFC values had been drawn from a population with a mean value of 0. From here, 325 T-statistics were retrieved (one for each unique edge), and the same procedure was repeated for each of the 10000 surrogate datasets, obtaining 10000 × 325 T* values. The difference of 1 minus the proportion of times each original T was larger than its corresponding 10000 T* statistics was considered the test *p*-value.

### Statistical analyses

The putative association between metastability and AD diagnose was tested by means of a multinomial logit model, using diagnose as independent variable in


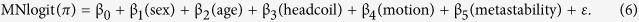


Given the distribution of headcoils in this study (Table 1), β_3_’s regressor was as a dummy variable with a value of “1” for 8-channel data and “0” for both HDNV and HNS. In-scanner motion was adjusted for using regressor β_4_. This analysis was implemented using Python’s *statsmodels* module^87^,^88^.

Additionally, between-diagnose differences in phase-based dynamic connectivity were tested by means of the network-based statistic (NBS) approach^89^. Briefly, NBS allows the statistical examination of potential differences in edge weights across different groups or conditions. It controls the family-wise error rate when statistical tests are conducted at single edges comprising a whole graph, on the basis of conventional cluster-based thresholding of statistical parametric maps. In the present scenario, the input matrices (graphs) represent the dFC measures between pairs of ICs; thus, potential dynamic connectivity disruptions are assessed at the edge level using NBS. The design matrices used for NBS here included adjustments for potential confounders (gender, age, headcoil and in-scanner motion); they were submitted to ANCOVA with the standard NBS parameters (5000 permutations; threshold: *p* = 0.05). Also, when appropriate, logistic regression tests were conducted individually on each of the 325 unique IC pairs. Some data were visualized using R’s PerformanceAnalytics package^90^,^91^.

## Additional Information

**How to cite this article**: Córdova-Palomera, A. *et al*. Disrupted global metastability and static and dynamic brain connectivity across individuals in the Alzheimer’s disease continuum. *Sci. Rep.* **7**, 40268; doi: 10.1038/srep40268 (2017).

**Publisher's note:** Springer Nature remains neutral with regard to jurisdictional claims in published maps and institutional affiliations.

## Supplementary Material

Supplementary Material

## Figures and Tables

**Table 1:** Clinical and demographic information.

	Age		MMSE score		Years of education		Sample size (n)	
	*Mean (SD*)	*Range*	*Mean (SD*)	*Range*	*Mean (SD*)	*Range*	*male/female*	
**AD**	71.5 (7.9)	53–91	22.8 (4.8)	5–30	12.4 (3.1)	7–19	35/45	
**MCI**	63.5 (11.1)	42–85	28.2 (2.1)	20–30	14.3 (2.8)	9–20	26/18	
**SCI**	63.6 (9.6)	49–84	29.4 (0.6)	28–30	15 (3.3)	9–22	12/14	
***Between-group differences** (**test statistics***)								
	***t***	***p***	***t***	***p***	***t***	***p***	***Χ***^***2***^	***p***
**ADvsMCI**	4.6	9.5e-6*	−6.6	1.1e-9*	−3.3	0.001*	2.1	0.148
**ADvsSCI**	4.2	6.7e-5*	−7	2.2e-10*	−3.6	4.5e-4*	1.7e-4	0.989
**MCIvsSCI**	−0.04	0.968	−2.9	0.005*	−0.9	0.351	0.6	0.423

Notes: Between-group differences were assessed with either two-tailed t-tests (continuous variables) or chi-square (*Χ*^*2*^, contingency tables degree of freedom). Abbreviations: SD, standard deviation; *statistically-significant p-value.
