# Supplementary material for: Disrupted global metastability and static and dynamic brain connectivity across individuals in the Alzheimer’s disease continuum
Source: Sci Rep. 2017 Jan 11;7:40268. doi: 10.1038/srep40268 (PMC5225495; doi:10.1038/srep40268)
Supplement: Supplementary Material [file srep40268-s1.pdf]

Supplementary material

The next material refers to the manuscript:

Disrupted global metastability and static and dynamic brain connectivity across individuals in the Alzheimer's disease continuum

Authored by:

Aldo Cordova-Palomera[a,], Tobias Kaufmann[a], Karin Persson[b,c], Dag Alnæs[a], Nhat Trung Doan[a], Torgeir Moberget[a], Martina Jonette Lund[a], Maria Lage Barca[b,c], Andreas Engvig[a,d], Anne Brækhus[b,c,e], Knut Engedal[b,c], Ole A. Andreassen[a], Geir Selbæk[b,f,g], Lars T. Westlye[a,h]*

[a] NORMENT, KG Jebsen Centre for Psychosis Research, Division of Mental Health and Addiction, Oslo University Hospital & Institute of Clinical Medicine, University of Oslo, Norway

[b] Norwegian National Advisory Unit on Ageing and Health, Vestfold Hospital Trust, Tønsberg, Norway

[c] Department of Geriatric Medicine, The Memory Clinic, Oslo University Hospital, Norway

[d] Department of Medicine, Diakonhjemmet hospital, Oslo, Norway

[e] Department of Neurology, Oslo University Hospital, Oslo, Norway

[f] Faculty of Medicine, University of Oslo, Oslo, Norway

[g] Centre for Old Age Psychiatric Research, Innlandet Hospital Trust, Ottestad, Norway

[h] Department of Psychology, University of Oslo, Oslo, Norway

* Corresponding author: Aldo Cordova-Palomera, Ph.D., email: aldoc@medisin.uio.no, postal address: Oslo University Hospital, PoBox 4956 Nydalen, 0424 OSLO, Norway, Telephone: +47 23 02 73 50, Fax: +47 23 02 73 33.

Figure S1. Pearson’s correlation coefficients of sFC and dFC measures

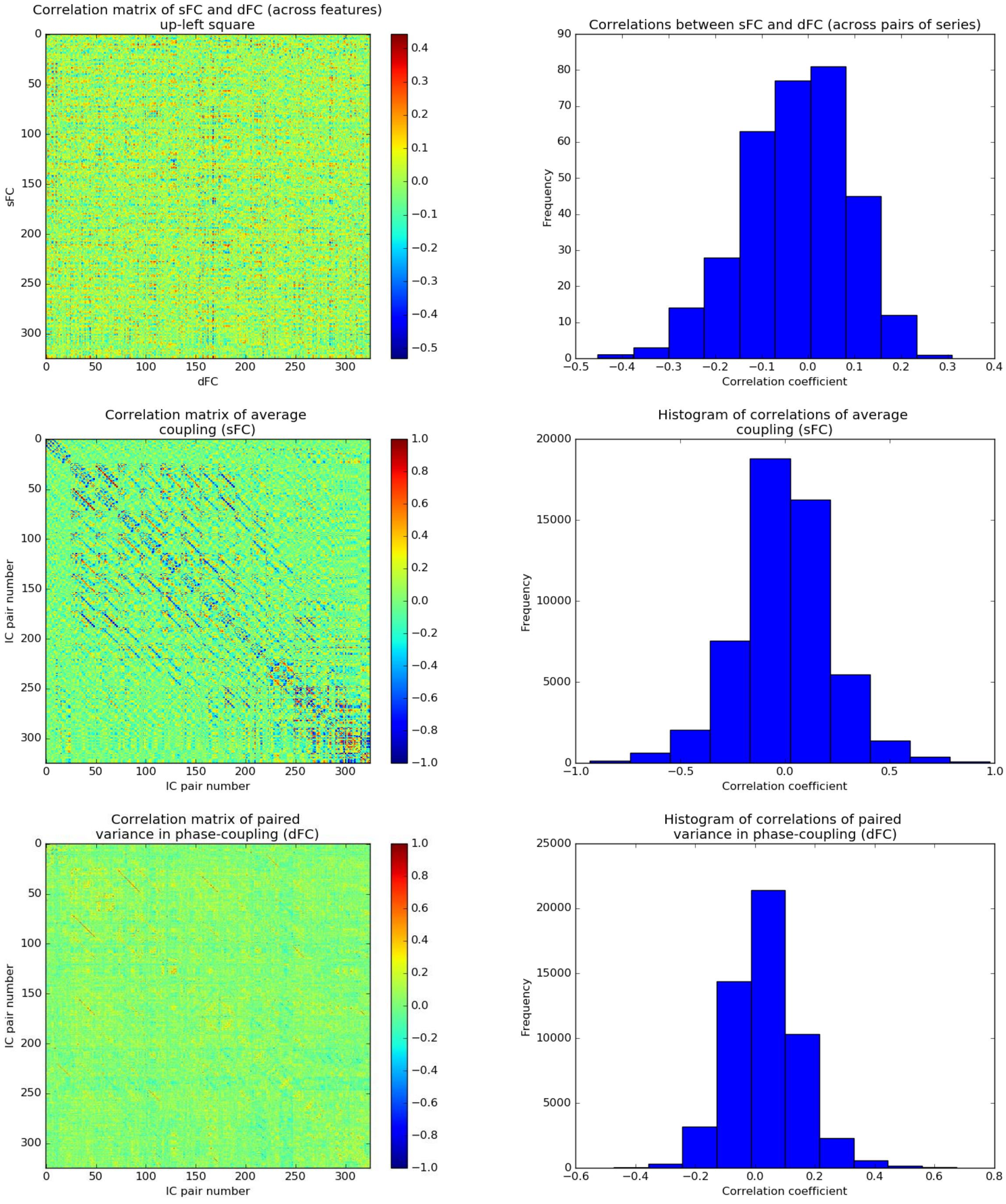

Figure S2. Relationship between temporal signal-to-noise ratio (tSNR) and diagnosis*metastability

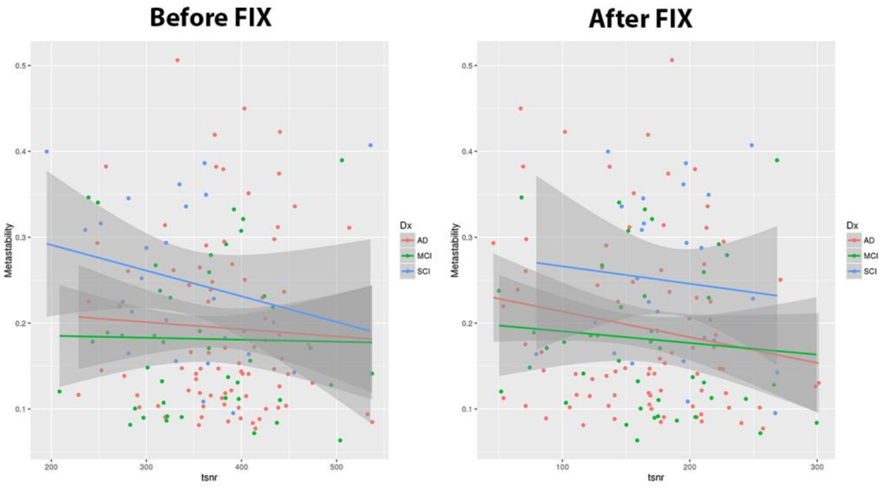

Figure S3. Relationship between metastability across diagnoses and subject-specific motion parameters measured after FIX and motion correction

Abbreviations: MCrelmean, movement relative to previous timepoint, as measured with MCFLIRT; NvarAbs, overall (absolute) variance (%) removed from the data; nrICs, number of ICs; nrNICs, number of “noisy” ICs; NvarRel, variance (%) removed, relative to the total variance explained by the ICs; propN, proportion of noise (%); meta, metastability.

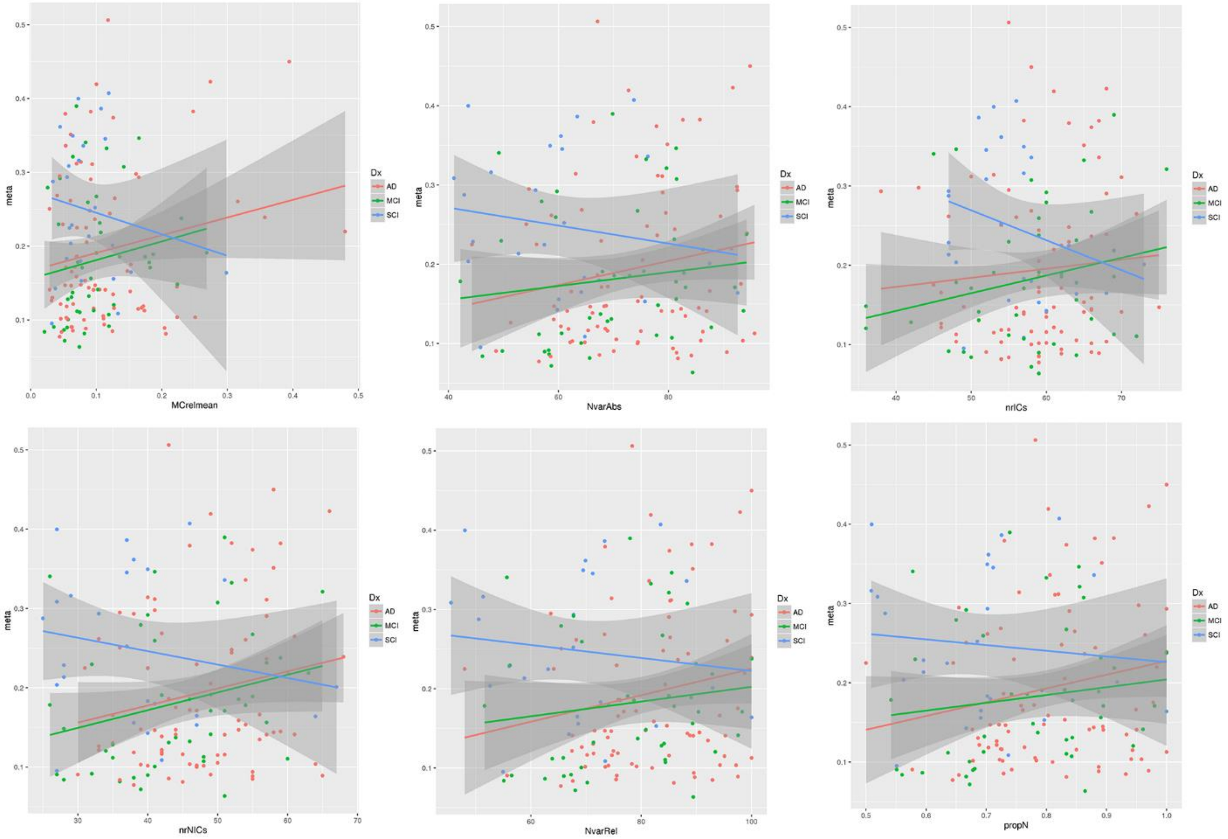

Figure S4. Estimated mean relative in-scanner head motion measured with FSL's MCFLIRT (before pre-processing)

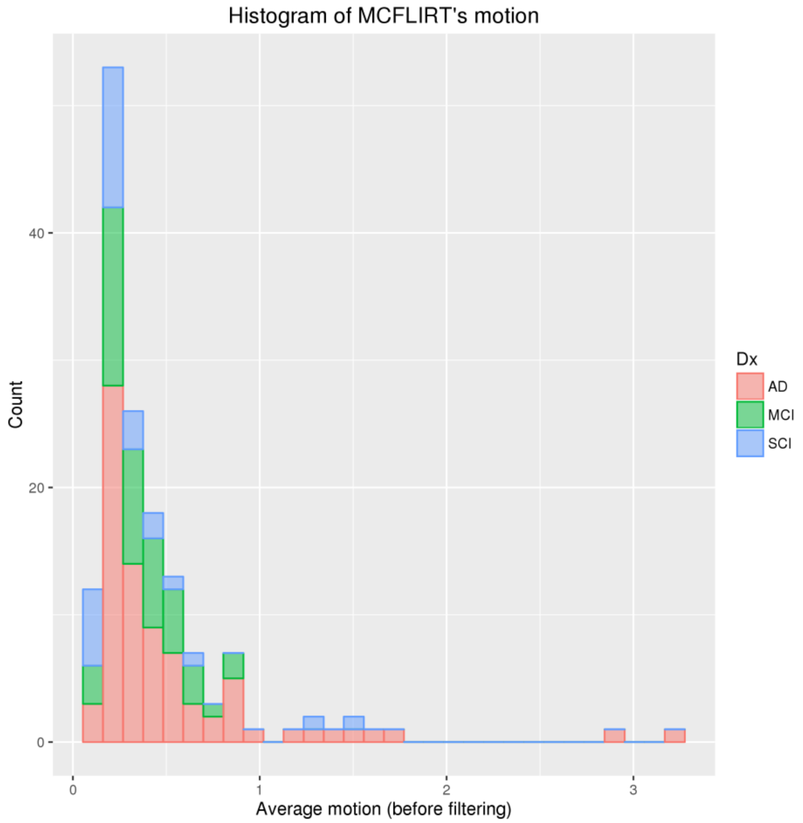

Table S1. Pre-processing and motion correction parameters across diagnostic groups

Abbreviations: nrICs, number of ICs; nrNICs, number of "noisy" ICs; propN, proportion of noise (%); NvarRel, variance (%) removed, relative to the total variance explained by the ICs; NvarAbs, overall (absolute) variance (%) removed from the data; RMSmotion, raw in-scanner motion, as measured by MCFLIRT, before filtering; MCrelmean, movement relative to previous timepoint, as measured with MCFLIRT, after filtering; tSNR, temporal signal-to-noise-ratio (original: before pre-processing; filtered: after pre-processing).

	**AD**		**MCI**		**SCI**		***Group comparison (Kruskal-Wallis tests)***	
Metric	***Mean (SD)***	***Range***	***Mean (SD)***	***Range***	***Mean (SD)***	***Range***	X^2	***p***
nrICs	59 (7.2)	38-75	57.6 (9)	36-76	55.5 (6.6)	47-73	6.5	0.039
nrNICs	47.8 (9)	30-68	44.4 (10.4)	26-65	38.6 (10.8)	25-67	17	0.0002
propN	0.8 (0.1)	0.5-1	0.8 (0.1)	0.5-1	0.7 (0.1)	0.5-1	18	0.0001
NvarRel (%)	81.6 (11.3)	48-100	77.6 (12.8)	51.6-100	68.2 (13.8)	45.5-100	18.9	$8*10^{-5}$
NvarAbs (%)	73.7 (12.2)	44.2-95.7	70.4 (13.4)	42.2-94.2	60.2 (13.4)	41-92.6	18	0.0001
RMSmotion	0.52 (0.54)	0.1-3.21	0.37 (0.19)	0.12-0.86	0.35 (0.35)	0.1-1.55	6.2	0.046
MCrelmean	0.12 (0.08)	0.03-0.5	0.1 (0.06)	0.02-0.27	0.09 (0.05)	0.03-0.3	1.7	0.421
tSNR original	380.9 (63.3)	228.6-537.4	361.4 (77.5)	208.7-537.2	342.3 (74.2)	195.3-535.6	7.6	0.022
tSNR filtered	163.9 (59)	45.8-301.2	165.4 (58.7)	50.3-299.6	187 (45)	79.6-268.6	2.7	0.259
